# Allokation knapper medizinischer Ressourcen auf COVID-19-PatientInnen. Ergebnisse einer Vignettenstudie

**DOI:** 10.1007/s11553-021-00909-x

**Published:** 2021-10-25

**Authors:** Julia Schmidt, Peter Kriwy

**Affiliations:** 1 Institut für Klinische Epidemiologie und Biometrie (IKE-B), Julius-Maximilians-Universität Würzburg, Josef-Schneider-Straße 2, 97080 Würzburg, Deutschland; 2 Institut für Soziologie, Technische Universität Chemnitz, Thüringer Weg 9, 09126 Chemnitz, Deutschland

**Keywords:** Entscheidungsverhalten, Ressourcenknappheit, Gerechtigkeit, Vignettenstudie, Medizinische Laien, Decision making behavior, Resource scarcity, Justice, Factorial survey, Medical laypersons

## Abstract

**Ziel der Studie:**

In der vorliegenden Studie werden Knappheitsentscheidungen von medizinischen Laien bei der Behandlung von COVID-19(„Coronavirus Disease 2019“)-Patienten untersucht.

**Methodik:**

In den multivariaten clusterkorrigierten Regressionen werden die Antworten auf 1802 Fallsituationen einer Vignettenstudie ausgewertet, die von 181 medizinischen Laien eingeschätzt wurden.

**Ergebnisse:**

Jüngere Patienten, Männer, Erkrankte mit hohen Genesungschancen, eigenen Kindern und einem Beruf in einem Krankenhaus werden von den medizinischen Laien bevorzugt eingestuft.

**Schlussfolgerung:**

Medizinische Laien wenden bewusst oder unbewusst soziale Kriterien der Entscheidungsfindung bei Knappheit der medizinischen Ressourcen an, die aus professioneller medizinischer und ethischer Sicht unzulässig sind. Zur Einschätzung der Akzeptanz in der allgemeinen Bevölkerung sollte dieser Umstand berücksichtigt werden.

## Abstract (en)

**Aim of the study:**

The present study investigated scarcity decision-making by medical laypersons in the treatment of COVID-19 (“Coronavirus Disease 2019”) patients.

**Methods:**

In the multivariate cluster-corrected regressions, the responses to 1802 case situations of a factorial survey assessed by 181 medical laypersons were evaluated.

**Results:**

Younger patients, men, those with a high chance of recovery, who have their own children and a job in a hospital were given priority by medical laypersons.

**Conclusion:**

Medical laypersons consciously or unconsciously apply social criteria of decision-making when medical resources are scarce, which are inadmissible from a professional medical and ethical point of view. In order to assess acceptance in the general population, this circumstance should be taken into account.

## Einleitung

Die SARS-CoV-2(„severe acute respiratory syndrome coronavirus 2“)-Pandemie stellt weltweit Gesundheitssysteme vor enorme Herausforderungen [12]. Die Zahl der an COVID-19(„Coronavirus Disease 2019“)-Erkrankten steigt ab März 2020 auch in Deutschland stark an. Bereits ab diesem Zeitpunkt beginnt eine Debatte über die Zuteilung von Ressourcen, für den Fall, dass nicht mehr alle erkrankten Personen adäquat versorgt werden können. Professionelle Akteure im Gesundheitswesen sind dem Gleichbehandlungsgrundsatz verpflichtet und dürfen somit keine ungleichen Zugänge (beispielsweise nach Alter, Geschlecht, Nationalität oder sozialem Status) zu medizinischen Ressourcen ermöglichen [7, 8]. Medizinische Laien sind auf Gleichbehandlung in dieser Hinsicht nicht geschult. Deswegen ist es von besonderem Interesse zu erforschen, welche Einstellungen medizinische Laien zur Ressourcenallokation im Kontext der SARS-CoV-2-Pandemie haben.

In der Diskussion um Knappheit in der Behandlung von COVID-19-Patienten werden strukturelle Ähnlichkeiten zum Verteilungsproblem im Bereich der Organtransplantation gesehen [11, 33]. Eine vollständige Gleichbehandlung von Bedürftigen ist unter Knappheit allerdings nicht möglich, insofern kann man keine vollständig wertfreien Ansätze in der Allokation erwarten [15]. Allgemein werden bei Knappheit medizinischer Ressourcen folgende Prinzipien diskutiert: Losverfahren, „first come first served“, Bedürftigste oder Jüngste zuerst, Maximierung verbleibender Lebensjahre oder Reziprozität [25].

Inwiefern die Allokation von Ressourcen von medizinischen Laien als gerecht eingeschätzt wird, hängt auch von individuellen Merkmalen der bewertenden Person ab, wie beispielsweise sozioökonomischer Status, Geschlecht, Alter, Lebensstil, Beruf oder Gesundheitsstatus [20].

Die theoretischen Überlegungen greifen Mechanismen auf der Ebene der Merkmale der Betroffenen auf (Vignettenhypothesen). Das Prinzip der Jüngsten zuerst, als ein prominentes Allokationsprinzip, sieht eine Allokation nach dem Alter vor. Junge Menschen sollen demnach bevorzugt behandelt werden, da sie noch mehr Lebensjahre vor sich haben und somit stärker von lebensrettenden Maßnahmen profitieren [19]. Das Argument, dass jeder ein Recht auf eine große Anzahl verbleibender Lebensjahre haben sollte, spielt an dieser Stelle eine entscheidende Rolle. Ältere Personen haben diese bereits verbraucht, während jüngeren Personen das Mehr an Lebensjahren durch eine Priorisierung bei der Allokationsentscheidung zugesprochen werden kann [32]. Demzufolge werden älteren Personen weniger Ressourcen zugesprochen, um das knappe Gut für jüngere Erkrankte aufwenden zu können. Wir stellen daher folgende Vignettenhypothese auf:

### HV1.

Mit steigendem Alter der an COVID-19 erkrankten Person, sinkt die Priorisierung bei der Vergabe knapper medizinischer Ressourcen.

Beim Allokationsprozess zu Spenderorganen ist die Erfolgsaussicht neben der Dringlichkeit nach dem Transplantationsgesetz (TPG) das einzig zulässige Kriterium, das den Wartelistenplatz der Bedürftigen beeinflusst [30]. Dieses Prinzip wird auf Knappheitsszenarien von COVID-19-Patienten übertragen:

### HV2.

Je höher die Genesungschancen der an COVID-19 erkrankten Person, desto höher die Priorisierung bei der Vergabe knapper medizinischer Ressourcen.

Die dritte Hypothese greift den Mechanismus gesellschaftsbezogener Reziprozität auf. In der Vergangenheit aufgebrachte Leistungen zur Schonung von Ressourcen oder Beiträge zum Aufrechterhalten von Kollektivgütern sollten demnach belohnt werden [21]. Erziehungsarbeit kann auch als Beitrag zu einem Kollektivgut verstanden werden [24]. Demzufolge sollten Personen mit Kindern im Allokationsprozess bevorzugt werden.

### HV3.

An COVID-19 erkrankte Personen mit Kindern werden im Vergleich zu kinderlosen Erkrankten bei der Vergabe knapper medizinischer Ressourcen priorisiert.

Am 30.03.2020 veröffentlichte das Bundesministerium für Arbeit und Soziales eine Liste systemrelevanter Bereiche, welche u. a. Beschäftigte in Krankenhäusern und im Groß- und Einzelhandel umfasst [6]. In den Medien entsteht in diesem Zusammenhang eine Debatte darüber, dass sowohl Personen im medizinischen Bereich, als auch im Versorgungssektor mehr Wertschätzung erfahren sollten [27]. Da diese Personengruppen vermehrt dem Kontakt zu anderen Menschen ausgesetzt sind, steigt ihr Infektionsrisiko. Ähnlich dem Mechanismus gesellschaftlicher Reziprozität kann dieses Verhalten instrumentellen Werteorientierungen zugeschrieben werden, die zukünftige gesellschaftliche Erträge sichern [21].

### HV4.

Personen aus systemrelevanten Bereichen werden bei der Vergabe knapper medizinischer Ressourcen priorisiert.

Der letzte Mechanismus, der auf Ebene der erkrankten Personen diskutiert wird, bezieht sich auf das Ausmaß der Knappheit. Rationale Akteure müssten bei steigender Knappheit sparsamer mit Ressourcen umgehen [29]. Aus dieser Überlegung folgt die letzte Vignettenhypothese:

### HV5.

Je knapper die Ressourcen, desto geringer die Priorisierung der an COVID-19 erkrankten Personen.

Die Hypothesen werden mit der Datengrundlage eines faktoriellen Surveys (Vignettenanalyse) überprüft.

## Methodik

Faktorielle Surveys werden häufig verwendet, um latente Einstellungsmuster von Befragten zu erfassen. Das Umfragedesign findet in diversen Bereichen Anwendung, beispielsweise in Bezug auf eine faire Einkommensverteilung [1, 16, 17], bei der Messung von Einstellungen zu abweichendem Verhalten (z. B. Fahren unter Alkoholeinfluss [2]) oder gesundheitsbezogenen Themen [13, 22, 28]. Bei Vignettenstudien werden den Befragten multidimensionale Situationsbeschreibungen vorgelegt. Die Variation der Ausprägungen der Dimensionen erfolgt zufällig, sodass auf diese Weise die experimentelle Logik in die konventionelle Umfrageforschung integriert werden kann [3]. Faktorielle Erhebungen eignen sich zudem gut zur Ermittlung von Evaluationsstrategien in komplexen Situationen, da diese in der Regel als realistischer empfunden werden als die isolierte Beantwortung einzelner Items [26] und sozial erwünschtes Antwortverhalten weniger stark ausgeprägt ist. Vignettenanalysen und hierzu verwandte methodische Zugänge via experimentell variierten Erhebungsinstrumenten haben sich auch zur Analyse von Gerechtigkeitsaspekten bei der Allokation von Spenderorganen bewährt [9, 13, 23].

In dem ersten Teil des Fragebogens der vorliegenden Studie wurden die Befragten aufgefordert zu bewerten, in welchem Umfang sie fiktiven Patienten behandlungsnotwendige Ressourcen zusprechen würden. Um zu verhindern, dass Befragte eigene Interpretationen treffen, was mit dem Begriff „Ressourcen“ bezeichnet wird, wurde in einem einleitenden Text der Studie erläutert, dass mit Ressourcen „medizinisches Personal, Krankenhausbetten, Beatmungsgeräte usw.“ gemeint sind. Zudem wurde in den Vignetten darauf hingewiesen, dass es sich immer um Situationen handelt, in denen zu wenige Ressourcen für eine umfassende Behandlung aller Patienten vorhanden sind, um zu verdeutlichen, dass es sich um ein Szenario handelt, in dem eine Priorisierung notwendig ist. Es wurden kurze Erzählungen entwickelt, welche Patienten beschreiben, die an COVID-19 erkrankt und auf eine Behandlung angewiesen sind. Den beschriebenen Personen wurde ein Geschlecht zugeordnet und ein jeweils variierender, neutral klingender Nachname verliehen (Müller, Hoffmann, Weber etc.), um den Vignettentext authentischer wirken zu lassen. Die Dimensionen mit dazugehörigen Ausprägungen sind in Tab. 1 veranschaulicht.

**Tab. 1:** Vignettenstruktur

Vignettendimension	Ausprägungen	Vignettentext
*Geschlecht*	Herr	Bauer
	Frau	
*Alter (Jahre)*	25	… Jahre, hat sich mit dem Coronavirus infiziert und hat einen schweren Krankheitsverlauf
	45	
	65	
	85	
*Genesungschancen*	Sehr hoch	Die Ärzte schätzen ihre/seine Genesungschancen bei einer umfassenden Behandlung als … ein
	Mittelmäßig	
	Eher gering	
*Kinder*	Keine Kinder	Sie/er hat …
	Ein Kind	
	Zwei Kinder	
*Berufsstatus*	In einem kaufmännischen Beruf	Und arbeitet …
	Im Supermarkt	
	Im Krankenhaus	
*Ressourcenknappheit*	Eine andere Person	Neben ihr/ihm brauchen noch … die gleiche Behandlung, es gibt jedoch nur Kapazitäten für eine Behandlung
	5 andere Personen	
	10 andere Personen	

Quelle: eigene Darstellung

Aus den sechs Dimensionen mit den jeweiligen Faktorausprägungen ergibt sich ein Vignettenuniversum von 2 × 4 × 3 × 3 × 3 × 3 = 648 möglichen Kombinationen. Aus diesen wurde eine zufällige Auswahl von 100 Kombinationen gezogen, welche in 10 Sets à 10 Vignetten aufgeteilt wurden. Die Anzahl der gewählten Dimensionen liegt im optimalen Bereich für die kognitive Verarbeitungsfähigkeit durchschnittlicher Befragter [4, 5]. Innerhalb der Sets wurden lediglich Reihenfolgeeffekte manuell ausgebessert, z. B. wenn mehrere Vignetten zu einem Geschlecht hintereinander präsentiert (gezogen) worden wären.

**Abb. 1:**
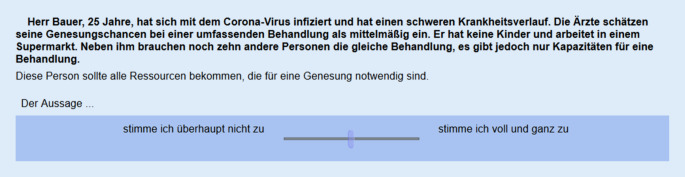
Beispielvignette mit Antwortskala. (Quelle: Ausschnitt aus dem eigenen Erhebungsinstrument)

Die Abb. 1 zeigt beispielhaft eine Vignette mit der zugehörigen Antwortskala, die von „stimme ich überhaupt nicht zu“ bis „stimme ich voll und ganz zu“ reicht. Der Schieberegler steht zunächst in der Mitte und wird leicht transparent angezeigt. Er musste von den Befragten angeklickt bzw. bewegt werden, um einen gültigen Wert zu erzeugen. Die Voreinstellung der Mittelposition signalisiert zumindest tendenziell, dass auch die nicht vollständige Ressourcenallokation eine legitime Antwortmöglichkeit darstellen kann. Befragte zeigen beim Vorliegen einer sog. Normierungsvignette, welche eine eher „schlechte“ Einstufung zum Gegenstandsbereich vornimmt, ein weniger sozial erwünschtes Antwortverhalten [13]. Durch Anklicken oder Verschieben des Reglers erzeugen Befragte Werte zwischen 0 und 100.

Der zweite Teil des Fragebogens ist der Personenfragebogen, der in jedem Set identisch ist. Hier wurden verschiedene soziodemografische Aspekte der Befragten abgefragt. Da im Rahmen der Studie das Allokationsverhalten von medizinischen Laien untersucht werden soll, wurde neben Alter und Geschlecht erfasst, ob es Teilnehmer gibt, welche in einem medizinischen oder pflegerischen Beruf arbeiten. Ebenso wurden alle Befragten gebeten anzugeben, ob sie selbst oder eine bzw. mehrere Personen aus dem näheren Umfeld aktuell oder in der Vergangenheit mit SARS-CoV‑2 infiziert waren, um auszuschließen, dass mögliche Effekte auf eigene Erfahrungen mit der COVID-19-Erkrankung zurückzuführen sind. Zudem wurde nach dem höchsten Bildungsabschluss, der subjektiven Schichteinstufung, Partnerschaft und dem Vorhandensein eigener Kinder gefragt.

Die Rekrutierung der Befragten erfolgte im Mai und Juni 2020 über zwei E‑Mail-Verteiler. A) Den Pool befragungsbereiter Studierender der Fakultät für Human- und Sozialwissenschaften der Technischen Universität Chemnitz und B) den Pool befragungsbereiter Personen der Sektion Medizin- und Gesundheitssoziologie der Deutschen Gesellschaft für Soziologie. Über den ersten Verteiler wurden eher jüngere Probanden angesprochen und über den zweiten Verteiler wurden eher ältere Befragte rekrutiert. Da das Zufallselement bereits im Ergebungsinstrument integriert ist, muss bei Vignettenstudien nicht primär auf eine Zufallsauswahl von Befragten geachtet werden [4]. Es ist hinreichend, wenn Varianz bezüglich der Hypothesenvariablen auf Personenebene gesichert ist.

Insgesamt haben 214 Personen den Online-Fragebogen vollständig ausgefüllt, wobei für den vorliegenden Auswertungszweck nur die gültigen Antworten medizinischer Laien (*n* = 181) verwendet werden. Eine detaillierte Beschreibung der Verteilung von Befragtenmerkmalen findet sich in Tab. 2.

**Tab. 2:** Verteilung der Befragtenmerkmale der multivariaten Analyse

Variable	*n*	Mean	SD	Minimum	Maximum
Alter	181	31,90	13,24	18	72
Bildungsjahre	181	13,92	3,08	9	20
Frau	181	0,66		0	1
Betroffenheit	181	0,19		0	1

Quelle: Eigene Daten, eigene Berechnung

*SD* Standardabweichung

Weiblich sind 66 % der Befragten. Das Durchschnittsalter der Befragten liegt bei 32 Jahren mit einer Standardabweichung (SD) von 13,2. Auch wenn mehr Frauen als Männer befragt wurden, so sind 62 Männer dennoch völlig hinreichend, um auf Geschlecht zu kontrollieren. Ähnliches gilt für die Altersverteilung. Das Durchschnittsalter von 32 Jahren ist zwar geringer als in allgemeinen Bevölkerungsbefragungen, jedoch wird mit der SD von 13,2 ebenfalls eine hinreichende Varianz im Alter erzeugt. Bildung variiert zwischen 9 (qualifizierter Hauptschulabschluss) und 20 (Promotion) Bildungsjahren und weist einen Mittelwert von 13,9 Bildungsjahren auf. 18 % der Befragten der multivariaten Analyse haben einen Hauptschulabschluss oder mittlere Reife. Unter den Befragten sind 18 Personen mit einem medizinischen oder pflegerischen Beruf. Da für die folgenden Hypothesentests die Einstellungen medizinischer Laien relevant sind, werden diese 18 Personen für die multivariaten Analysen ausgeschlossen. Bei der verbleibenden Stichprobe werden weitere 15 Personen nicht berücksichtigt, weil fehlende Werte bei Alter (6×), Gender (1×), Bildungsjahren (9×) und Betroffenheit (2×) vorliegen. Zudem wurden die subjektive Schichteinstufung [14], Partnerschaft und Vorhandensein eigener Kinder erfragt, die in den Analysen vor dem Hintergrund sparsamer Modellierung nicht berücksichtigt werden.

Da aufgrund des Vignettendesigns die Unabhängigkeitsannahme der Elemente verletzt ist (eine befragte Person beantwortet 10 Vignetten), wird auf Befragtenebene eine clusterkorrigierte Regression durchgeführt [4]. Alle Berechnungen wurden mit STATA 14 (StataCorp, 4905 Lakeway Drive, College Station, TX, USA) durchgeführt.

## Ergebnisse

Der Mittelwert der abhängigen Variablen liegt bei 58,47 für 1802 von medizinischen Laien bewertete Vignetten. Die Verteilung ist symmetrisch (Skewness −0,26) und geringfügig flacher als eine Normalverteilung (Kurtosis 2,12). Insgesamt wurden alle 101 Ausprägungen des Wertebereichs von 0–100 genutzt, mit einer leichten Häufung an beiden Extremwerten. Die daraus resultierende leichte Zweigipfligkeit ist für die Regressionsanalyse jedoch kein Problem, da der gesamte Wertebereich der abhängigen Variablen von den Befragten genutzt wurde und zugleich Symmetrie vorliegt.

Mittels einer multiplen linearen Regression wird analysiert, inwiefern Faktoren auf Vignetten- und Personenebene einen Einfluss auf das Bewertungsverhalten der Befragten haben. Für das nachfolgend beschriebene Regressionsmodell kann Linearität zwischen abhängiger und metrischen unabhängigen Variablen, das Fehlen von Multikollinearität, Homoskedastizität der Residuen sowie die Unabhängigkeit der Residuen zwischen den Clustern (Befragten) angenommen werden, sodass davon ausgegangen werden kann, dass bestmögliche Schätzwerte erreicht werden, welche die BLUE-Eigenschaften vorweisen. In das Modell werden alle Vignettendimensionen sowie die Kontrollvariablen Geschlecht, Alter, Bildung in Bildungsjahren und Erfahrungen mit COVID-19 (Erkrankung selbst durchlebt oder im persönlichen Umfeld vorgekommen) verwendet.

Hypothese 1 wird vorläufig bestätigt, da mit steigendem Alter der fiktiven erkrankten Personen die Priorisierung bei der Zuweisung von medizinischen Ressourcen statistisch signifikant sinkt. Im Vergleich zu den 25-jährigen erkrankten Personen sinkt die Zuweisebereitschaft bei 45-Jährigen um 3,5 Prozentpunkte. Bei 85-Jährigen sinkt die Priorisierung um statistisch höchst signifikante 22 Prozentpunkte im Vergleich zur den jungen fiktiven Erkrankten.

Mit steigenden Genesungschancen sollte, Hypothese 2 zufolge, die Priorisierung zunehmen. Dies bestätigt sich vorläufig mit einem statistisch höchst signifikanten Zusammenhang. Mittlere Genesungschancen erhöhen die Priorisierung um 13,4 Prozentpunkte und sehr hohe Chancen auf Gesundung um 20,8 Prozentpunkte.

Ebenfalls statistisch signifikant häufiger werden Personen, welche ein oder zwei Kinder haben bei Allokationsentscheidungen bevorzugt. Die Effektgröße unterscheidet sich dabei kaum zwischen der Anzahl der Kinder, somit wird auch Hypothese 3 vorläufig bestätigt.

Im Krankenhaus beschäftigte Personen werden im Vergleich zu Personen mit kaufmännischen Berufen statistisch signifikant häufiger benötigte Ressourcen zugesprochen. Zwischen kaufmännischen Angestellten und Supermarktmitarbeitern gibt es jedoch keinen Unterschied, obwohl Mitarbeitende im Lebensmittelhandel ebenfalls als systemrelevant gelten, analog zu Beschäftigten im Krankenhaus. Wenn der medizinische Beruf gegen den Beruf im Supermarkt getestet wird (Berechnung mit Beruf im Supermarkt als Referenzkategorie, nicht in Tab. 3 enthalten), dann werden Personen mit medizinischem Beruf auch gegenüber Mitarbeitenden im Supermarkt priorisiert. Hypothese 4 wird somit spezifiziert auf den Krankenhaussektor vorläufig bestätigt.

**Tab. 3:** OLS-Regression mit Clusterkorrektur, AV: Zuweisung aller zur Genesung notwendigen Ressourcen (Spektrum: 100 „stimme voll zu“ bis 0 „stimme nicht zu“)

	Reg.-Koeff.	t‑Wert
**Vignettenvariablen**		
*Mann*	2,34	2,27^a^
*Ref. Alter*: 25 Jahre	–	–
45 Jahre	−3,46	−2,31^a^
65 Jahre	−8,65	−5,57^c^
85 Jahre	−21,98	−10,41^c^
*Ref. Genesung*: eher gering	–	–
Mittel	13,40	7,67^c^
Sehr hoch	20,77	9,96^c^
*Ref. Kinder*: kein Kind	–	–
Ein Kind	7,81	5,28^c^
Zwei Kinder	7,93	5,61^c^
*Ref. Beruf*: kaufmännischer Bereich	–	–
Krankenhaus	4,41	3,26^b^
Supermarkt	1,22	0,91
*Ref. Knappheit*: 5 andere Personen	–	–
1 andere	3,77	2,36^a^
10 andere	−1,07	−0,84
**Befragtenvariablen**		
Alter (10 Jahre)	2,31	2,64^b^
Frau	4,55	1,77
Bildungsjahre	−0,69	−1,86
Betroffenheit	−1,34	−0,41
*Konstante*	45,96	6,85^c^

Quelle: Eigene Daten, eigene Berechnung

Vignetten: *n* = 1802, Personen: *n* = 181 (ohne Personen mit medizinischem Beruf), R^2^: 0,22

*OLS* Ordinary Least Squares

^a^*p* < 0,05

^b^*p* < 0,01

^c^*p* < 0,001

Mit steigender Knappheit sollte, Hypothese 5 zufolge, die Priorisierung sinken. Es zeigt sich, dass geringfügige Knappheit statistisch signifikant um 3,8 Prozentpunkte mit erhöhter Zuweisebereitschaft medizinischer Ressourcen einhergeht. Es ist dabei weitgehend unerheblich, welche der beiden anderen Kategorien als Referenzkategorie verwendet wird. Nimmt man 10 andere bedürftige Erkrankte als Referenzkategorie (nicht in Tab. 3 dargestellt), so erhält man das ähnliche Ergebnis von 4,8 Prozentpunkten erhöhter Zuweisebereitschaft. Die Knappheitshypothese wird somit vorläufig bestätigt.

Obwohl zum Geschlecht der fiktiven Vignettenpersonen keine Hypothese generiert wurde, ist dennoch ein statistisch signifikanter Zusammenhang zu berichten. Männer erhalten eine um 2,3 Prozentpunkte höhere Priorisierung als Frauen.

Bei den Kontrollvariablen hat nur das Lebensalter der Befragten einen signifikanten Effekt. Je höher das Alter der Befragten, desto höher die Priorisierung medizinischer Ressourcen. Die weiteren Kontrollvariablen (Geschlecht, Bildung in Bildungsjahren und Kenntnis von COVID-19-Erkrankungen im eigenen sozialen Umfeld) haben keinen statistisch signifikanten Einfluss auf die Priorisierung knapper medizinischer Ressourcen. Wobei dazugesagt werden sollte, dass der Effekt der Bildungsjahre mit einem t‑Wert von −1,86 nur knapp das 5 %-Niveau verpasst.

## Diskussion

In der vorliegenden Vignettenstudie wurden die Antworten von 181 Personen ausgewertet, was 1802 bewerteten Fallsituationen entspricht. Zuvor wurden 18 Befragte mit einem Beruf aus dem medizinischen Bereich für die Analysen ausgeschlossen, da die Allokationseinstellungen von medizinischen Laien im Forschungsinteresse stehen. In den Vignetten wurden fiktive, an COVID-19 erkrankte Personen präsentiert und deren Geschlecht, Alter, Genesungschancen, Vorhandensein eigener Kinder, Beruf und Knappheit der medizinischen Ressourcen experimentell variiert.

Die fünf theoretisch hergeleiteten Hypothesen werden vorläufig bestätigt. Mit steigendem Alter, höheren Genesungschancen, wenn eigene Kinder vorhanden sind, ein systemrelevanter Beruf im Krankenhaus ausgeübt wird (Angestellte im Supermarkt erfahren keine Priorisierung) und bei geringer Knappheit der zur Verfügung stehenden Ressourcen weisen die Befragten den fiktiven erkrankten Personen vermehrt medizinische Ressourcen für die Behandlung zu. Auch wenn zum Geschlecht der Erkrankten keine Hypothese formuliert wurde ist zu berichten, dass Männer geringfügig aber statistisch signifikant bevorzugt werden. Im Vergleich zu Frauen zeigen Männer allgemein eine schwächere oder zu starke Immunreaktion bei Erkrankung an COVID-19 auf. Beide Mechanismen erhöhen das Sterberisiko bei Männern [31], deswegen könnte es sein, dass Männer aus diesem Grund eine leichte Bevorzugung erhalten.

Die Ergebnisse fügen sich gut in den vorliegenden Forschungsstand ein, der in ähnlichen Knappheitssituationen von multiplen zugrunde liegenden Mechanismen der Allokationsentscheidungen ausgeht. Sowohl das Prinzip der „Jüngsten zuerst“ als auch utilitaristische Ideen und die Belohnung von gesellschaftlichem Nutzen werden über die Ressourcenzuweisung der befragten medizinischen Laien abgebildet und finden sich auch in etwa bei Allokationseinstellungen im Zusammenhang mit der Verteilung knapper Spenderorgane [9, 13, 23].

Da das Konzept der Genesungschancen bei COVID-19-Patienten sehr ähnlich ist zur Erfolgsaussicht beim Allokationsprozess zu Spenderorganen (s. § 12 Abs. 3 Transplantationsgesetz), könnte man dieses Allokationskriterium durchaus als „zulässiges“ oder „legitimes“ Kriterium bei der Zuweisung von Ressourcen zur Behandlung von COVID-19-Patienten durch medizinische Laien bezeichnen. Es ist jedoch zu diskutieren, inwiefern Genesungschancen in der medizinischen Praxis überhaupt belastbar zeitnah beziffert werden können. Schwieriger wird die Bewertung der Entscheidungssituation jedoch bei der Diskriminierung nach dem Alter der Bedürftigen und nach dem Vorhandensein von Kindern. Dies mag aus alltagsweltlicher Sicht eine mögliche Daumenregel darstellen, aus professioneller medizinischer und juristischer Sicht wären Entscheidungsregeln dieser Art nicht zulässig. Für die Einschätzung der Akzeptanz von Knappheitsentscheidungen in der Bevölkerung ist es jedoch von hoher Wichtigkeit zu wissen, dass medizinische Laien solche Mechanismen der Entscheidungsfindung bewusst oder unbewusst anwenden.

Limitationen der vorliegenden Studie sind ebenfalls zu diskutieren. Aufgrund der Rekrutierungsstrategie sind höhergebildete Befragte überrepräsentiert (der Anteil von Personen mit Haupt- und Realschulabschluss liegt bei 18 % in der Vignettenstudie vs. 35 % in der Allgemeinbevölkerung [10]). Da jedoch der Einfluss der Bildungsjahre in den vorliegenden multivariaten Berechnungen nur knapp nicht auf dem 5 %-Niveau signifikant ist (t-Wert −1,86), könnte vermutet werden, dass eine höhere Fallzahl bei gering gebildeten Befragten einen signifikanten Effekt mit sich bringen könnte. Im Vergleich zu anderen deutlichen Resultaten der Studie (z. B. der Effekt bei hochaltrigen Erkrankten oder solchen mit sehr hohen Genesungschancen), spielen etwaige Einflüsse einiger Bildungsjahre von Befragten mehr oder weniger kaum eine Rolle. Neben der Bildung gemessen in Bildungsjahren wurden auch Dummy-Variablen der Abschlüsse geprüft und dabei Haupt- und Realschulabschlüsse zusammengefasst (zusammen 32 Befragte). Es ergeben sich auch für diese Messvariante keine Zusammenhänge zwischen Bildung und Outcome, d. h. die Abwesenheit eines signifikanten Bildungseffekts mit vorliegender Fallzahl kann als robustes Resultat gewertet werden. Insofern ist diese deskriptive Schieflage kein Problem für die Qualität der Hypothesentests.

Da die soziodemographischen Variablen hinreichend Varianz aufweisen, sollte externe Validität gegeben sein. Auch bezogen auf die interne Validität liegt kein offensichtliches Verdachtsmoment vor, denn das Vignettenuniversum war nicht zu groß und die Anzahl an Dimensionen und Ausprägungen pro Vignette haben die empfohlenen Grenzwerte nicht überschritten [4].

Insgesamt wurden 214 Personen befragt, davon haben 181 Personen, die für die multivariaten Analysen 1802 beantworteten Vignetten produziert. Eine hinreichende Analysepower sollte dementsprechend vorliegen. Diese Schlussfolgerung wird auch durch die Anzahl vorläufig bestätigter Hypothesen gestützt.

Die vorliegenden Ergebnisse sind dabei behilflich, Knappheitsentscheidungen in der medizinischen Versorgung aus der Sicht medizinischer Laien zu verstehen. Nicht medizinisch geschulte Menschen machen in der Zuweisung knapper Ressourcen deutliche Unterschiede nach sozialen Kriterien, deren Anwendung in der professionellen medizinischen Praxis undenkbar wäre. Ein gerecht empfundener Ressourcenumgang zeichnet sich durch Gleichheit in der Entscheidungspraxis aus. International betrachtet gilt somit in den meisten Ländern der Grundsatz „keine Diskriminierung“ bei COVID-19-Triage [18]. Diese Aspekte könnten in der allgemeinen medialen Kommunikation verstärkt aufgegriffen werden, um eine möglichst hohe Akzeptanz der Ressourcenallokation im Zusammenhang mit COVID-19 zu erzielen.

Zukünftige Forschung könnte auch die Befragung spezieller Populationen hinsichtlich ihrer Einstellungen bezüglich der Ressourcenallokation in den Fokus nehmen (z. B. Juristen, Ärzte oder ältere Personen). Die Durchführung von faktoriellen Surveys im internationalen Vergleich könnte zeigen, ob in unterschiedlichen Ländern andere Einstellungsmuster vorherrschen. Auch wäre es sehr interessant herauszufinden, ob bestimmte Allokationsentscheidungen COVID-19-Besonderheiten sind. Hierzu könnten Knappheitsentscheidungen bei der Behandlung von Personen mit COVID-19 im Vergleich mit Einstellungen zur Behandlung von anderweitig erkrankten Personen verglichen werden.

Ein der Vignettenanalyse ähnliches Verfahren sind Discrete-choice-Experimente. Solche Experimente könnten hilfreich sein, speziell zum vorliegenden Thema die Vignettenergebnisse erneuten Tests zu unterziehen. Da sich bei diesem alternativen Ansatz die Befragten bei ihrer Priorisierung zwischen zwei Personen oder Situationen entscheiden müssen, ist die „übergangene“ Alternative für die Befragten explizit präsent, während in der Vignettenstudie nur die Besonderheiten der beschriebenen Person aufgezeigt werden. Ähnlichkeiten der Ergebnisse mit unterschiedlichen empirischen Zugängen würde die Belastbarkeit der Resultate stärken.

Inwiefern solche Erkenntnisse das Potenzial haben, bei der Erstellung klinisch-ethischer Empfehlungen im Kontext medizinischer Ressourcenknappheit berücksichtigt zu werden, könnten ebenfalls zukünftige Forschungsprojekte aufgreifen.

## Fazit für die Praxis

- Für die Einschätzung der Akzeptanz von Knappheitsentscheidungen in der Bevölkerung ist es wichtig zu wissen, dass medizinische Laien nach Alter und dem Vorhandensein eigener Kinder der erkrankten Personen etc. diskriminieren, obwohl die Anwendung solcher Mechanismen der Entscheidungsfindung aus professioneller Sicht unzulässig ist.
- Das Konzept der Genesungschancen bei COVID-19(„Coronavirus Disease 2019“)-Patienten ist sehr ähnlich zum Konzept der Erfolgsaussicht beim Allokationsprozess von Spenderorganen, das laut Transplantationsgesetz eines der wenigen zulässigen Allokationskriterien darstellt. Medizinische Entscheidungen von Ärzten in Knappheitssituationen könnten sich gegebenenfalls u. a. daran orientieren.
