# Erratum zu: Allokation knapper medizinischer Ressourcen auf COVID-19-PatientInnen. Ergebnisse einer Vignettenstudie

**DOI:** 10.1007/s11553-021-00922-0

**Published:** 2021-11-16

**Authors:** Julia Schmidt, Peter Kriwy

**Affiliations:** 1 Institut für Klinische Epidemiologie und Biometrie (IKE-B), Julius-Maximilians-Universität Würzburg, Josef-Schneider-Straße 2, 97080 Würzburg, Deutschland; 2 Institut für Soziologie, Technische Universität Chemnitz, Thüringer Weg 9, 09126 Chemnitz, Deutschland

**Erratum zu:**

**Präv Gesundheitsf 2021**

10.1007/s11553-021-00909-x

In der ursprünglichen Version war im zweiten Punkt des Fazits für die Praxis geschrieben: „Das Konzept der Genesungschancen bei COVID-19(‚Coronavirus Disease 2019‘)-Patienten ist sehr ähnlich zum Konzept der Erfolgsaussicht beim Allokationsprozess von Spenderorganen, das laut Transplantationsgesetz eines der weniger zulässigen Allokationskriterien darstellt.“ Korrekt ist die Formulierung „(…) das laut Transplantationsgesetz eines der wenigen zulässigen Allokationskriterien darstellt.“

Zudem war im ursprünglichen Text ein Satz zur Feldarbeit enthalten: „(…) Pool befragungsbereiter Studierender der XY Fakultät der XY Universität (Fachbereich für Begutachtung anonymisiert) (…)“ Dieser Teil wurde geändert in „(…) Pool befragungsbereiter Studierender der Fakultät für Human- und Sozialwissenschaften der Technischen Universität Chemnitz (…)“.

Der Originalbeitrag wurde korrigiert.

